# Clinical Characteristics of Primary Orthostatic Tremor – a Comprehensive Clinical Assessment of Patients in Sweden

**DOI:** 10.5334/tohm.1143

**Published:** 2026-03-05

**Authors:** Karolina af Edholm, Mathias Sundgren, Erik Fransén, Henrik Sjöström, Anders Svenningsson

**Affiliations:** 1 Department of Clinical Sciences, Karolinska Institutet Danderyds Hospital, Entrévägen 2, 182 88 Danderyd, Sweden; 2 Department of Clinical Neuroscience, Karolinska Institutet, Stockholm, Sweden; 3 Department of Neurology, Karolinska University Hospital, Stockholm, Sweden; 4 School of Electrical Engineering and Computer Science, KTH Royal Institute of Technology, Stockholm, Sweden; 5 Digital Futures, KTH Royal Institute of Technology, Stockholm, Sweden; 6 Science for Life Laboratory, KTH Royal Institute of Technology, Stockholm, Sweden; 7 Centre for neurology, Academic specialist centre, Stockholm, Sweden; 8 Department of Clinical Sciences, Karolinska Institutet Danderyd Hospital, Stockholm, Sweden

**Keywords:** Primary orthostatic tremor, Shaky legs, Tremor

## Abstract

**Background:**

Primary orthostatic tremor (POT) is a rare neurological disease presenting as a bilaterally coherent tremor of 13–18 Hz and a subjective sensation of unsteadiness while standing. Patients are severely affected by the inability to stand and often eventually referred to walking aids and dependence on others.

**Objectives:**

This study aimed to investigate the clinical characteristics of POT in a Swedish patient population by interviews and questionnaires.

**Methods:**

Patients with POT were recruited nationwide in Sweden. All participants underwent neurological examination, structured interview and evaluation according to nine standardized rating scales and questionnaires, including the novel orthostatic tremor scales OTIP and OT-10.

**Results:**

Fifty-two participants with EMG-verified POT were included in the final analysis. Disease duration was not significantly correlated to disease severity, while OTIP and OT-10 were highly correlated with severity of POT. Postural or action tremor in the arms were present in 58%. Mild signs of parkinsonism were common, and the combination of mild rigidity and bradykinesia was present in 25%. Symptoms of depression and anxiety were present in 25%. Although quality of life was often severely affected, 65% performed activities of daily life independently.

**Discussion:**

Patients with POT may be severely affected by their disease, independently of disease duration. Multiple associated symptoms like tremor in the upper extremities and mild Parkinsonian features need to be recognized by healthcare professionals.

**Highlights:**

52 patients with POT were clinically examined with rating scales and questionnaires. Additional symptoms like other tremors, parkinsonian signs, and depressive symptoms were common. Disease severity did not correlate to additional symptoms or disease duration. Questionnaires like OTIP and OT-10 can be recommended for assessment of disease severity.

## Introduction

Primary orthostatic tremor (POT) is a rare neurological disorder characterized by a high-frequency, almost invisible, tremor while standing [1]. The condition was first described by Pazzaglia et al. in 1970, and the term “orthostatic tremor” was introduced by Heilman et al. in 1984 [2,3]. There is no data of prevalence or incidence, but the condition is considered rare and diagnosis is often delayed due to unawareness of its existence and difficulties in visually detecting the tremor [4,5].

Clinical characteristics in patient populations with POT have previously been described in several small studies, only a few larger studies have included more than 40 patients [4,6,7,8,9,10]. However, data collection has often been reduced to compilation of medical records and retrospective patient-reported data, why a systematic assessment of both physical and psychological signs could further improve the understanding of POT. The disease is often described as progressive and patients are troubled by the fear of falling and dreading the long-term outcome of increasing imbalance, even though evidence supporting a correlation between disease severity and disease duration is weak [4,5,9,10,11]. The unique constellation of clinical symptoms of POT underscores the need for specialized assessment tools to capture the full spectrum of symptoms and patient-reported difficulties. Recently, two assessment scales for POT have been developed, the Orthostatic Tremor Severity and Disability Scale (OT-10) and the Orthostatic Tremor Impact Profile (OTIP) [11,12].

In this study, patients with POT were systematically assessed with interviews and questionnaires, including the OT-10 and OTIP, and neurological examination to evaluate disease severity. Questionnaires and rating scales covering areas of related symptomatology like tremor, parkinsonism, quality of life, depression and cognitive impairments were also included.

## Methods

### Participants

Patients with POT visiting or referred to the two largest neurology clinics in Stockholm (Danderyd Hospital and Karolinska University Hospital) during the period of September 2020 to April 2025 were invited to participate in the study. Patients from other regions of Sweden who contacted study investigators were also invited. Information about the study was disseminated through news articles in Swedish medical journals and an official presentation of the study on the university website. Inclusion criteria were a diagnosis of orthostatic tremor confirmed by a neurologist and tremor frequency of 13 to 18 Hz verified by EMG. Exclusion criteria were secondary orthostatic tremor where other neurological disorders could be defined and suspected to be causally related to the orthostatic tremor. The study was approved by the Swedish Ethical Review Authority. All participants provided written informed consent in accordance with the Declaration of Helsinki.

### Clinical parameters

All included patients were neurologically examined and interviewed by KaE about medical history, heredity, effect of tremor medications, tremor symptom onset, ability to stand and walk, the use of walking aids, education, regular physical exercises, alcohol and tobacco, sleep quality, exposure to possible harmful substances (pesticides, solvents, heavy metals) and previous head trauma. Relevant exposure of harmful substances was defined as regular exposure for more than five years. For the assessment of tolerated time in standing, participants estimated the interval of time they would tolerate standing during a usual day, for example when standing in a queue in the grocery store. If they found it difficult to precise this period of time, they would be asked whether they could stand less than 30 seconds, up to one minute, up to two minutes, up to five minutes, up to ten minutes, or more than ten minutes.

### Rating scales

Acquisition of all data from questionnaires and rating scales was done by KaE live in front of the patients. Questionnaires and rating scales about tremor included Fahn Tolosa Marín Tremor Rating Scale (FTM TRS), Orthostatic Tremor Impact Profile (OTIP), and Orthostatic Tremor Severity and Disability Scale (OT-10) [11,12,13]. FTM TRS was developed to mainly assess tremor in upper extremities and consists of 21 items including both physical examination, practical tests (writing, drawing, pouring water) as well as anamnestic questions of how tremor affects aspects of everyday life (0–144 points), rating of each item can gain 0–4 points where 0 is normal. OTIP is a questionnaire developed to cover symptoms of POT where patients can gain maximum 188 points, where part 1–4 include physical, social and everyday life impairments (0–112 points) and part 5 emotional impact (0–76 points), rating goes 0–4 where 0 is normal. OT-10 is also a questionnaire developed to cover POT symptoms consisting of ten items covering mainly physical impairment related to POT (0–50 points), rating 0–5 per item where 0 is normal.

The rating scale used for assessing parkinsonism was Unified Parkinson’s Disease Rating Scale (UPDRS) part I, II and III [14]. Part I and II consists of 17 questions and cover anamnestic motor- and non-motor symptoms rating 0–4 where 0 is normal (0–68 points). Part III covers present motor symptoms of parkinsonism and was rated by a trained assessor (0–100 points).

Independence and quality of life was assessed with the Swedish versions of Schwab and England ADL (Activities of Daily Living) Scale and the EuroQol EQ-5D-3L (EQ5D and EQ VAS) measurement of health-related quality of life [15,16]. Schwab and England ADL Scale assess capabilities in patients with impaired mobility on a scale of 100% to 0%, where 100% is completely independent and unaware of difficulties related to the disease, and 0% is almost fully comatose and completely dependent. EQ5D is a self-rated assessment of health status in five areas concerning mobility, self-care, usual activities, pain/discomfort and anxiety/depression. The patient chooses a level of “no problems”, “some problems” or “extreme problems” in each area. EQ VAS is a visual analogue scale of self-rated health, where 100 is best possible health, and 0 is worst possible health.

Depression and anxiety were assessed with the Swedish versions of Montgomery-Asberg Depression Rating Scale (MADRS) and Hospital Anxiety and Depression Rating Scale (HADS) [17,18]. MADRS is a self-rated scale including 9 items that can generate 0–6 points (total of 54 points). HADS is a patient-reported self-rated 14 item scale where item response is rated on a 4-point scale (0–3). Seven of the items concern anxiety symptoms and seven concerns depression. The anxiety part and depression part can attain 0–21 points each.

Cognitive function was assessed with the Swedish version of Montreal Cognitive Assessment (MoCA), a screening tool for cognitive impairment where patients can attain 0 to 30 points, where 26 points or more is considered normal [19]. An additional point is usually given when there is an educational level of 12 years or less, however, in this study results are presented without any additional point. Patients were assured not to have previously completed the MoCA test closer than 6 months prior to the test date.

### Data Analysis

The analysis of clinical data, and results from questionnaires and rating scales were exploratory. Results are reported as percent or mean, standard deviation, median and range, or as percentage of the participants. To investigate the relationship between disease severity and results from questionnaires and rating scales Pearson correlation analyses were performed. A p-value of 0.05 was considered significant. After Bonferroni correction the threshold for significance level was adjusted to 0.0025. This approach ensures rigorous statistical evaluation while controlling the risk of false-positive findings in multiple testing situations.

## Results

### Participants

60 participants were initially included in the study between September 2020 and April 2025. All participants had previously been examined for tremor by a neurologist and received the diagnosis orthostatic tremor. Upon inclusion, medical records were reviewed for confirmation of tremor frequency and results from brain imaging and laboratory tests that might indicate other causes of tremor. In total eight participants were excluded from the final analysis. Two participants did not fulfil the criteria of POT due to slow frequency and irregular tremor activity when examined later during the study period. Three participants were examined with brain MRI later during the study period and diagnosed with unexpected findings that could influence symptoms and prognosis (multiple cerebellar infarctions; extensive white matter changes and cerebellar atrophy; cortical atrophy indicating Corticobasal Degeneration). Two participants were diagnosed with Parkinson’s disease and Lewy Body Dementia, respectively, during the study period. One participant presented a left sided chorea, negative for excessive CAG-repeats in the Huntingtin gene, and without progression during the full study period. The remaining 52 participants all presented rhythmic tremor in standing position with a frequency of 13–18 Hz and no other neurological disorder that could be related to the orthostatic tremor. 24 participants were living outside of the Stockholm area and were included after they had voluntarily contacted the involved researchers.

### Clinical characteristics

Interviews, compilation of clinical data, and clinical ratings were performed by principal investigator K af Edholm, board certified in neurology. Clinical data is presented in Table 1. The participants’ age, sex, disease duration, time to diagnosis and heredity were consistent with data from previous studies [4,6,7,8,9,10]. Ethnicity was mainly Caucasian, and the majority had a college or university education. Alcohol consumption as well as tobacco use was generally low. A majority had either been exposed to previous head trauma, harmful substances or been drinking water from a private well for longer periods (>five years). The most common comorbidity was cardiovascular disease, followed by orthopaedic conditions and thyroid disease (current hypothyroid 17%, previous thyroid disease/goitre 8%). Neurological conditions (migraine, Restless legs, REM-sleep behaviour disorder, stuttering, trigeminal neuralgia, childhood epilepsy, previous meningitis, previous Guillan-Barré Syndrome, congenital facial hemiparesis) were reported in 23.1%. Depression or anxiety was previously diagnosed in 13.5%. A full list of comorbidities is presented in supplementary table 1.

**Table 1:** Clinical characteristics.


CLINICAL CHARACTERISTICS	MEAN	SD	MEDIAN	RANGE	%

Age	67.7	10.7	70	34–83	

Disease duration	11	7.1	10	1–29	

Years to diagnosis	7	6.3	4.5	1–21	

Females					73%

Males					27%

Caucasian					98%

Asian					2%

First degree relative with orthostatic tremor					7.7%

First degree relative with other tremor disorder					7.7%

First degree relative with other neurodegenerative disorder					15.4%

Up to 12 years education					19.6%

Vocational education					11.5%

University/college education					69.2%

No present alcohol use					13.5%

Low alcohol use (<10 stdgl/w)					71.2%

High alcohol use (>10 stdgl/w)					15.4%

No tobacco use					42.3%

Previous tobacco use					48.1%

Present tobacco use					7.7%

No exposure to harmful substances, head trauma or private well					42.3%

Exposure to harmful substances (pesticides, solvents, heavy metals)					21.2%

History of mild to moderate head trauma					19.2%

Regular drinking from private well					21.2%


Clinical characteristics of patients with POT. SD = standard deviation. Stgl/w = standard glasses/week.

### Disease severity

Participants were asked to assess their ability to stand and walk, Table 2. Patients frequently reported that their standing ability was highly fluctuating between days and situations, where stress reduced tolerated standing time and a safe environment increased it. The majority reported feeling mostly unrestricted while walking and could walk more than 5 km without difficulties related to their POT. Two participants could not stand without support at all, and walk less than 100 m related to POT, and they were also using wheelchairs in their daily life. The majority were not using any walking aid even though they had to adapt their daily life to their POT symptoms. 26.9% regularly used a walker or walking sticks for support. Correlation of disease severity to disease duration, age and the results from the rating scales and questionnaires are presented in Table 4. No significant correlation could be found between neither disease duration nor age when compared to any of the other parameters like disease severity or results from rating scales and questionnaires. There was no significant correlation between alcohol use, tobacco use, exposure, exercise or current medical tremor treatment with disease severity like tolerated standing time, the use of walking aid, walking distance or scores on OTIP or OT-10 (data not shown).

**Table 2:** Subjective reports of disease severity and treatments.


DISEASE SEVERITY					

WALKING AID		WALKING DISTANCE		TOLERATED TIME IN STANDING	

Wheelchair	3.8%	<100 m	3.8%	<30 seconds	17.5%

Walker	15.4%	0.1–1 km	15.4%	30–60 seconds	15.7%

Walking sticks	11.5%	1–2 km	3.8%	1–2 minutes	15.7%

None	69.2%	2–5 km	9.6%	2–5 minutes	33.3%

		>5 km	67.3%	5–10 minutes	5.9%

				>10 minutes	11.8%

**TREATMENTS**		**PARTICIPANTS (N)**		**POSITIVE EFFECT**	

Clonazepam		24		41.7%	

Gabapentin		24		45.8%	

Propranolol		26		42.3%	

Primidone		5		20%	

Perampanel		4		75%	

Valproic acid		4		0%	

Zonisamide		2		0%	

Dopaminergic medications		2		0%	

Deep Brain Stimulation		2		50%	


Disease severity presented as percentage of the study population using walking aids, reported maximum walking distance and reported tolerated time in standing. Previous and current treatments for orthostatic tremor presented as number of participants and percentage of participants reporting positive effect.

### Treatments

Propranolol, clonazepam and gabapentin were the most frequently current or previously used medications for POT (Table 2), and were all reported to have a positive effect at least in the beginning in 41–46% of the cases. Perampanel was/had been used by four participants and three had experienced a clear positive effect on the tremor, but side effects like depression and irritability were a recurrent problem. Five participants had tried primidone, only one experienced a positive effect on the tremor. During the study 31 participants (60%) were currently using tremor medications. Two participants had deep brain stimulation (DBS) in the ventral intermediate nucleus in thalamus, and one had initial positive effect.

### Rating scales

#### Tremor

Results from each questionnaire and rating scale are presented in Table 3. Both POT ratings scales, OTIP and OT-10, had large standard deviations and wide ranges in total scores, showing a wide diversity in symptom severity between patients. Both OTIP and OT-10 correlated significantly with tolerated standing time, the use of walking aids, and walking distance (p-values are presented in Table 4). They also correlated with independence in ADL (Schwab & England ADL scale), and self-rated health (EQ VAS). However, neither the results from OTIP or OT-10 correlated significantly with disease duration or age. The total OTIP score was also highly influenced by ratings of emotional impact in part 5 and had significant correlation with symptoms of depression/anxiety (MADRS and HADS).

**Table 3:** Rating scales and questionnaires.


RATING SCALES AND QUESTIONNAIRES				MEAN	SD	MEDIAN	RANGE

OTIP total (0–188)				52.8	34.3	43	0–144

OTIP part 1–4 (0–112)				31.3	24	24	0–102

OTIP part 5 (0–76)				20.9	16	18	0–54

OT-10 (0–50)				23.7	9.8	24	0–41

FTM TRS (0–144)				11.3	7.3	9	3–34

Head tremor		4%					

Tongue tremor		44%					

Postural arm tremor		48%					

Action/intention arm tremor		21%					

Postural leg tremor		33%					

Action/intention leg tremor		8%					

UPDRS I-II (0–68)				7.4	3.9	7	2–19

Reported falling <12 months		30%					

Freezing of gait		42%					

UPDRS III (0–100)				5.6	5.8	4	0–24

	Mild (1p)	Moderate (2–3p)	Severe (4p)				

Rigidity	25%	12%	0%				

Bradykinesia	37%	8%	0%				

Rest tremor	2%	0%	0%				

Postural instability	36%	15%	4%				

Schwab & England ADL scale (0–100%)				79.4	14	80	50–100

Independent in ADL (80–100%)		65%					

EQ5D VAS (0–100)				66.1	18	70	31–93

EQ5D	No problem	Some problem	Extreme problem				

Mobility	65%	35%	0%				

Hygiene	88%	10%	2%				

Daily activities	61%	33%	6%				

Pain/discomfort	19%	77%	4%				

Anxiety/depression	40%	56%	4%				

HADS (0–42)				8.4	6.3	7	0–27

Normal (anxiety 0–7p, depression 0–7p)		75%					

Anxiety (8–21p), no depression (0–7p)		13.5%					

Depression (8–21p), no anxiety (0–7p)		3.8%					

Anxiety (8–21p) and depression (8–21p)		7.7%					

MADRS (0–54)				8.5	6.4	8	0–25

Normal (0–12p)		76.5%					

Mild depression (13–19 p)		15.7%					

Moderate depression (20–34 p)		7.8%					

Severe depression (>35p)		0%					

MoCA (0–30)				26.4	2.7	27	19–30

Normal (26–30p)		69%					

Mild cognitive impairment (18–25p)		31%					


Results from rating scales and questionnaires presented as mean, SD, median, range and percentage of study population. *SD = standard deviation. OTIP = Orthostatic tremor impact profile. OT-10 = Orthostatic tremor Severity and disability scale. FTM TRS = Fahn Tolosa Marín tremor rating scale. S&E ADL = Schwab and England activities of daily life scale. EQ VAS = Euroqol visual analogue scale. HADS = Hospital anxiety and depression scale. MADRS = Montgomery Asberg depression rating scale. MoCA = Montreal cognitive assessment test*.

**Table 4:** Correlation analysis.


	DISEASE DURATION	AGE	STANDING TIME	WALKING AID	WALKING DISTANCE	OTIP TOTAL	OTIP 1-4	OTIP 5	OT-10

**Standing time**	0.01569	0.012173							

**Walking aid**	0.009334	0.004828	**0.000666**						

**Walking distance**	0.421807	0.003717	**0.000284**	**<.00001**					

**OTIP total**	0.499753	0.097781	**<.00001**	**0.000652**	**<.00001**				

**OTIP 1-4**	0.105749	0.006905	**<.00001**	**<.00001**	**<.00001**	**<.00001**			

**OTIP 5**	0.373048	0.716388	**0.001838**	0.956795	0.159408	**<.00001**	0.003389		

**OT-10**	0.148923	0.039986	**<.00001**	**<.00001**	**0.000096**	**<.00001**	**<.00001**	0.005633	

**FTM TRS**	0.007393	0.005084	0.008499	0.048743	0.313914	0.120183	0.041088	0.770201	0.061433

**UPDRS III**	0.043444	0.024341	0.373048	0.009527	0.386175	0.292759	0.011701	0.178927	0.03898

**S&E ADL**	0.140713	0.015525	**0.00022**	**<.00001**	**0.00014**	**<.00001**	**<.00001**	0.046563	**0.000017**

**EQ VAS**	0.354238	0.132856	**0.001171**	0.024728	0.012093	**<.00001**	**<.00001**	**<.00001**	**0.000125**

**HADS**	0.782076	0.503813	0.009441	0.406371	0.092686	**<.00001**	**0.000286**	**<.00001**	0.010542

**MADRS**	0.597691	0.960158	0.01008	0.975303	0.345419	**<.00001**	**0.002131**	**<.00001**	0.02321

**MoCA**	0.409997	0.089313	0.294074	0.100941	**0.000244**	0.097336	0.01633	0.981476	0.129498


Correlation between disease severity and results from rating scales and questionnaires. Pearson correlation and Bonferroni correction for multiple comparison. P-values presented were considered significant when p < 0.0025, marked as bold. *OTIP = Orthostatic tremor impact profile. OT-10 = Orthostatic tremor Severity and disability scale. FTM TRS = Fahn Tolosa Marín tremor rating scale. S&E ADL = Schwab and England activities of daily life scale. EQ VAS = Euroqol visual analogue scale. HADS = Hospital anxiety and depression scale. MADRS = Montgomery Asberg depression rating scale. MoCA = Montreal cognitive assessment test*.

A full list of results from FTM TRS is presented in supplementary table 2. Total scores were generally low, but a high number of patients had mild postural tremor in arms and legs, and mild action/intention tremor in the arms. 44% presented tremor or trembling of the tongue. The results from FTM TRS did not correlate with any of the other rating scales or questionnaires, and neither to disease severity nor duration.

#### Parkinsonian features

The total score in UPDRS was generally low both in part I-II and part III. 30% reported falling within the last twelve months, and 42% reported symptoms similar to freezing of gait due to difficulties of moving the legs after a period of standing. In part III, points were mainly gained from postural/action tremor, mild bradykinesia, mild rigidity and postural instability. A substantial part of participants (25%) had the combination of mild bradykinesia and mild rigidity, but after careful clinical examination and review of their medical records, none was considered to have Parkinson’s disease. There was no difference in age or disease duration in the group with bradykinesia and/or rigidity compared to the group without, but a slighter higher prevalence of men (32% vs 24%, p = 0.0006).

#### Independence and quality of life

**Figure 1:**
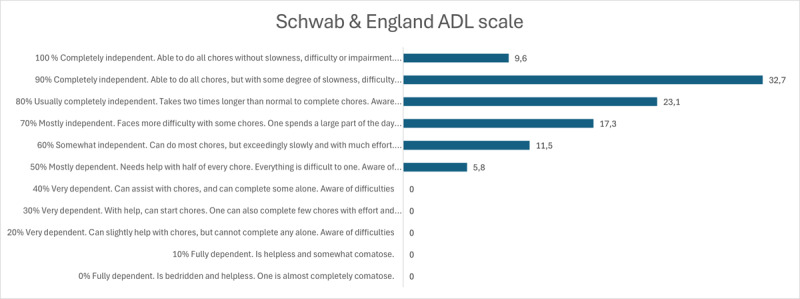
Patient-reported difficulties with activities of daily life according to Schwab & England Activities of Daily Life scale.

The Schwab and England ADL scale revealed that patients commonly reported awareness of difficulties and slowness while the majority were still completely independent (rating 80% or higher). Only 5.8% were mostly dependent, needed help with household chores and experienced difficulties with everything (Figure 1).

In EQ5D patients commonly reported problems related to mobility, daily activities, pain/discomfort and anxiety/depression, while self-care was less affected. In self-rated health measured with EQ VAS, the average score was 66. Results of EQ VAS did not correlate with age or disease duration.

#### Depression and anxiety

Results from the questionnaires for anxiety and depression, HADS and MADRS, were consistent in the assessment of depressive symptoms, where 75% and 76.5% respectively were in the normal spectrum. The anxiety-items of HADS generated higher scores than the depression-items indicating that anxiety constitutes a major part of affective symptoms in POT. Symptoms of depression and/or anxiety did not correlate with disease duration, or disease severity like tolerated time in standing, walking distance or walking aids (Table 4).

#### Cognitive function

The majority scored 26 or higher at the MoCA test, a level considered as normal. 31% scored 19–25 points, indicating mild cognitive impairment, but since age and education were factors not accounted for, the level of clinically relevant cognitive impairment may be lower. There was no significant correlation of MoCA score with age or disease duration, clinical characteristics or any of the other rating scales.

## Discussion

We here present data from one of the largest studies of patients with POT including both comprehensive clinical examination and a broad panel of questionnaires. We thereby characterize this patient group both clinically and through their perception how the disease impacts their physical and mental health. One of the main findings was that the OT-specific scales OTIP and OT-10 correlated strongly with both physical and mental consequences of the disorder, confirming these scales as important tools in the clinical follow-up of POT patients. On the other hand, neither disease duration or age correlated with disease severity measures such as tolerated time in standing, walking distance, use of walking aids, self-rated health, independence in ADL or depressive symptoms. This suggests that the progression of the disease is slow, and that individual factors play a large role in determining disease severity.

The clinical characteristics like age, sex, disease duration and family history were consistent with results from previous studies of POT [4,6,7,8,10]. The common presentation of arm tremor and depressive symptoms have been reported before [4,6,8,9]. Motor symptoms like arm tremor, mild bradykinesia and mild rigidity were not linked to faster disease progression. Specifically, these symptoms did not correlate with reduced tolerated time in standing nor increased need for walking aids. Mild bradykinesia, rigidity and tremor may also be related to ageing, and since we did not have a control group, we cannot exclude this could be normal findings related to age. There are also studies where the presence of Parkinsonian features like bradykinesia and rigidity have not been confirmed in the POT population [20]. In our study, there was no difference in age or disease duration in the group of participants with parkinsonian features but a slightly higher prevalence of men. According to the Consensus statement on tremors, POT is an isolated tremor syndrome, and the labelling “Primary orthostatic tremor plus” is referred to patients with an additional neurological condition (like Parkinson’s disease, spinocerebellar ataxia or dementia) [1]. However, the presence of arm tremor or mild Parkinsonian signs should not exclude the possibility of a POT diagnosis.

Tongue tremor was reported in 44%, but this number has to be taken with caution since it is very difficult to visually distinguish a rhythmical tongue tremor from trembling of the tongue caused be repeated motor impersistence. The presence of tongue tremor could suggest involvement of cranial muscles, which has been described previously in a minority of patients, and one participant even reported she could hear her tremor [21]. An unexpected high frequency of participants presented reported “freezing of gait” (42%) in our study. “Freezing of gait” was reported as a feeling of not being able to get the legs to move after a period of sitting or standing and has been described previously [22]. Even though “freezing of gait” mainly have been used for parkinsonian related gait difficulties, and the pathogenesis behind gait difficulties in POT may not be the same, this seems to be a relevant symptom to pay attention to while examining patients with POT.

Our study has the limitation of partly relying on patient-reported data, which may be influenced by recall bias. In the estimation of disease duration many participants, especially those with long disease duration, were unsure about exactly which year they noticed symptom onset. Symptom onset was also frequently described as a very slowly decreased tolerability of standing in crowded places where apparent shaking was a later phenomenon. Disease duration may have been estimated both shorter and longer than actual time span.

How to define disease severity in POT has not been clarified, although several previous studies have discussed this issue. Tolerated time in standing may be the obvious measurement, but this is also complicated to define. Participants reported that tolerated time in standing was difficult to estimate precisely since this was highly fluctuating between situations and most of the participants also experienced “good” and “bad” days. Participants were mainly explaining the tolerated time in standing as depending on how far they would push themselves when the feeling of instability and muscle exhaustion was increasing. The estimation of walking distance had similar potential drawbacks, and the use of walking aids may also be dependent on other factors such as orthopaedic conditions. The OT-10 scale has been validated for both internal consistency and test-retest reliability but to our knowledge there are no reports of its use in longitudinal studies of POT [12]. The OTIP scale was initially reported for a longitudinal study of 6 years, where the need for walking aids and reported falls both increased during this period, while the score of the OTIP scale did not [11]. Although both OT-10 and OTIP can be considered as useful for rating disease severity in POT, it is still unsure if they fully catch the patient reported subjective progression or the objective progression measurements like falls and the use of walking aids.

In our study affective symptoms were prominent in 25% of the participants, and anxiety was the main concern. These results align with the study of Bhatti and colleagues where patients with orthostatic tremor presented a high prevalence of depressive and especially anxiety spectrum disorders (24% with history of depression, 6.9% with ongoing major depression and 37.9% with agoraphobia) [23]. Maugest and colleagues have also reported a high prevalence of anxiety (41%) and depression (18%) among patients with POT, and how the fear of falling has the largest impact on the health-related quality of life [9]. Depression and anxiety may affect the participant’s interpretation of self-rated health in EQ5D and EQ VAS or independence in ADL. There may also be an increase of depressive symptoms as a result of disease severity but just like in the study of Maugest and colleagues, none of the results from the questionnaires for anxiety and depression correlated with disease duration. There was neither any correlation between decrease in cognitive function and disease duration or tolerated time in standing, indicating that progression in POT does not necessarily imply cognitive impairment.

## Conclusion

Patients with POT often show additional symptoms like arm tremor, tongue tremor, mild parkinsonian signs, and depressive symptoms, however this does not seem to correlate with a worse disease progression. Disease severity is rather related to a reduced independence in ADL and self-rated health, factors with impact on quality of life.

## Additional File

The additional file for this article can be found as follows:

10.5334/tohm.1143.s1 Supplementary file. Supplementary table 1 and Supplementary table 2.
