# Supplementary material for: Clinical Characteristics of Primary Orthostatic Tremor – a Comprehensive Clinical Assessment of Patients in Sweden
Source: Tremor Other Hyperkinet Mov (N Y). 2026 Mar 5;16:14. doi: 10.5334/tohm.1143 (PMC12962244; doi:10.5334/tohm.1143)
Supplement: Supplementary file. — Supplementary table 1 and Supplementary table 2. [file tohm-16-1-1143-s1.pdf]

Supplementary table 1.

**Comorbidity**	
Cardiovascular disease (hypertonia, atrial fibrillation, atrioventricular block, heart failure, previous cardiac infarction, aneurysm, claudicatio intermittens, previous perimyocarditis, previous deep vein thrombosis)	51.2%
Orthopaedic problems (osteoarthritis, musculoskeletal pain, osteoporosis, previous spinal disc hernia, spinal stenosis, previous fractures of the lower extremities)	34.6%
Thyroid disease (hypothyroid (n=9), previously treated for hyperthyroid/goitre (n=4))	25.0%
Neurological disease (migraine, Restless legs, stuttering, trigeminal neuralgia, childhood epilepsy, previous meningitis, previous Guillan Barré Syndrome, facial hemiparesis)	23.1%
Gastrointestinal disease (obstipation, previous cholecystectomy or appendectomy, Chron's disease, gastroesophageal reflux)	21.2%
Pulmonary disease (asthma, chronic obstructive lung disease, sleep apnoea, previous sever pneumonia with respiratory care)	15.4%
Psychiatric disease (depression, anxiety)	13.5%
Cancer (prostate, testicle, cervix, lymphoma, leukaemia)	9.6%
Diabetes (type 1 and 2)	7.7%
No comorbidity	5.8%

Comorbidities and percentage of participants.

Supplementary table 2.

**FTM TRS**	**1p (n)**	**2p (n)**	**3p (n)**	**4p (n)**	**1-4p (%)**
1 face rest tremor	1	0	0	0	1.9
2 tongue rest tremor	2	0	0	0	1.9
2 tongue postural tremor	23	0	0	0	44.2
3 voice action tremor	0	0	0	0	0
4 head rest tremor	0	0	0	0	0
4 head postural tremor	2	0	0	0	3.8
5 R arm rest tremor	0	0	0	0	0
5 R arm postural tremor	18	4	0	0	42.3
5 R arm action tremor	12	0	0	0	23.1
6 L arm rest tremor	1	0	0	0	1.9
6 L arm postural tremor	22	3	0	0	48.1

6 L arm action tremor	12	0	0	0	23.1
7 trunk rest tremor	1	0	0	0	1.9
7 trunk postural tremor	4	0	0	0	7.7
8 R leg rest tremor	0	0	0	0	0
8 R leg postural tremor	15	2	0	0	32.7
8 R leg action tremor	4	0	0	0	7.7
9 L leg rest tremor	0	0	0	0	0
9 L leg postural tremor	14	2	0	0	30.8
9 L leg action tremor	2	0	0	0	3.8
10 handwriting	8	2	0	0	19.2
11 Drawing A R	19	2	0	0	40.4
11 Drawing A L	36	7	0	0	82.7
12 Drawing B R	30	4	0	0	65.4
12 Drawing B L	29	17	2	0	92.3
13 Drawing C R	17	1	0	0	34.6
13 Drawing C L	22	5	4	0	59.6
14 Pouring R	14	1	1	0	30.8
14 Pouring L	14	2	1	0	32.7
15 Speaking	1	0	0	0	1.9
16 Feeding	4	1	0	0	9.6
17 Bringing liquids	8	1	0	0	17.3
18 Hygiene	27	2	1	0	57.7
19 Dressing	22	0	0	0	43.3
20 Writing	9	2	0	0	21.2
21 Working	16	14	10	0	76.9

Results from Fahn Tolosa Marín Tremor Rating Scale. R = right. L = left. Number and percentage of participants.
